# Relevance of the Spectral Analysis Method of Tilted Fiber Bragg Grating-Based Biosensors: A Case-Study for Heart Failure Monitoring

**DOI:** 10.3390/s22062141

**Published:** 2022-03-10

**Authors:** Miguel Vidal, Maria Simone Soares, Médéric Loyez, Florinda M. Costa, Christophe Caucheteur, Carlos Marques, Sónia O. Pereira, Cátia Leitão

**Affiliations:** 1 Physics Department & I3N, University of Aveiro, 3810-193 Aveiro, Portugal; miguelvidal@ua.pt (M.V.); msimone.fsoares@ua.pt (M.S.S.); flor@ua.pt (F.M.C.); carlos.marques@ua.pt (C.M.); sonia.pereira@ua.pt (S.O.P.); 2 Electromagnetism and Telecommunication Department, University of Mons, 31 Bld Dolez, 7000 Mons, Belgium; mederic.loyez@umons.ac.be (M.L.); christophe.caucheteur@umons.ac.be (C.C.)

**Keywords:** optical fiber sensors, surface plasmon resonance (SPR), spectral demodulation methods, cardiac biomarker, NT-proBNP, biosensors

## Abstract

Optical fiber technology has rapidly progressed over the years, providing valuable benefits for biosensing purposes such as sensor miniaturization and the possibility for remote and real-time monitoring. In particular, tilted fiber Bragg gratings (TFBGs) are extremely sensitive to refractive index variations taking place on their surface. The present work comprises a case-study on the impact of different methods of analysis applied to decode spectral variations of bare and plasmonic TFBGs during the detection of N-terminal B-type natriuretic peptide (NT-proBNP), a heart failure biomarker, namely by following the most sensitive mode, peaks of the spectral envelopes, and the envelopes’ crossing point and area. Tracking the lower envelope resulted in the lowest limits of detection (LOD) for bare and plasmonic TFBGs, namely, 0.75 ng/mL and 0.19 ng/mL, respectively. This work demonstrates the importance of the analysis method on the outcome results, which is crucial to attain the most reliable and sensitive method with lower LOD sensors. Furthermore, it makes the scientific community aware to take careful attention when comparing the performance of different biosensors in which different analysis methods were used.

## 1. Introduction

Over the years, biosensors have become an appealing alternative to conventional laboratory methods for analyte detection and quantification as they are cost-effective, easier to handle, of smaller dimensions and can provide a faster response. Biosensors consist of analytical devices capable of recognizing a specific analyte and converting an event into a measurable signal using a proper transducer. Such devices have been widely explored in a broad number of fields such as medical diagnosis, the food industry, drug delivery and environmental monitoring [1,2]. The biorecognition molecules employed depend on the intended application and are generally enzymes, antibodies/antigens or nucleic acids, being responsible for the biochemical interactions with the target that lead to physicochemical changes detectable by the transducer [3]. Among the existing biosensors, optical-fiber-based sensors stand out for analyte detection due to their highly sensitive, fast, remote and real-time sensing ability, allied with their biocompatibility, robustness, flexibility, immunity towards electromagnetic interference and small size, allowing for sensor miniaturization [4,5,6]. This way, these biosensors provide the opportunity to be used as point-of-care devices which can lead to improved healthcare [3,7].

For biosensing applications, it is imperative that the light propagating within the optical fiber is brought into contact with the surrounding medium. This can be achieved through different sensing structures such as unclad [8], D-shaped [9], tapered [10], end-face reflected [11] fibers and fiber gratings (namely, etched fiber Bragg gratings) [12], long period gratings [13] and tilted fiber Bragg gratings (TFBGs) [14]. TFBGs have proved to be a great biosensing tool, garnering high refractometric sensitivity and a low limit of detection (LOD), while providing benefits such as easy light injection, self-compensation of temperature oscillations and not requiring cladding removal, hence maintaining its mechanical strength [2,5,15]. The sensitivity can be enhanced by combining the TFBG with the surface plasmon resonance (SPR) effect, which is achieved through coating the fiber surface with a thin metallic layer. SPR denotes the oscillation of free electrons at a metal-dielectric interface stimulated by incident light [16], being highly sensitive to biochemical alterations on the interface [17]. This optical detection technique has been extensively used due to enabling highly sensitive and label-free sensing of molecules binding in real-time [18,19].

The transmitted spectrum of a TFBG is characterized by numerous narrow attenuation bands that represent coupling from the core mode to various cladding modes [20]. The spectral position of these resonances depends on the effective refractive index (RI) of the corresponding cladding mode and hence on the optical properties of the surrounding medium [18,21], such as the RI. As a result, the resonance wavelengths shift to larger values with the increase in the surrounding RI, while the intensity progressively decreases [22]. The deposition of a metal film, usually of gold (Au) owing to its biocompatibility and stainless properties, on the TFBG surface allows SPR excitation. This leads to the absorption of a fraction of the light energy in a certain wavelength, which varies according to the RI of the surrounding medium. This is expressed by a resonance attenuation in the transmitted spectrum [14,15].

Given the extensive number of resonances present in the TFBG spectrum, the data analysis of such sensors is still challenging, and hence different demodulation protocols have been proposed, resulting in distinct outcomes. The most common demodulation methods are based on wavelength and/or amplitude shifts of the most sensitive cladding modes, extracted from the lower peak of the mode [23]. For bare TFBGs, many studies have reported that the most sensitive modes belong to the cut-off region, which involves the cut-off mode and its vicinity. The effective RI of this mode is very close to the external medium’s RI, and hence its evanescent field possesses a deep infiltration beyond the fiber, making the corresponding resonance highly sensitive to external RI variations [17,24,25]. In addition to the shift in wavelength, as the surrounding RI increases and approaches the cladding modes’ effective RI, the coupling strength between the cladding modes and the core mode decreases, making them weakly guided and thus leads to an amplitude dip. In addition to the single mode method, the normalized area delineated by the lower and upper envelopes has also been applied for refractometry purposes. The normalized area is computed, in the spectral window of interest, as the ratio between the area covered by the lower and upper envelope curves for each RI value and the area of a reference spectrum, for example, in air. Considering the amplitude decrease, a smoothing of the spectrum is verified with increasing the surrounding RI that leads to a reduction in the normalized area [26].

In plasmonic TFBGs, such as gold-coated TFBGs (Au-TFBGs), while the SPR mode is considered the most sensitive mode [27,28], it is generally monitored upon large surrounding RI changes. For minor variations (in the order of 10^−4^ RIU) and thus small spectral shifts, it is very challenging to accurately monitor it due to its strong attenuation and broadening by the plasmon [15,24,27]. In alternative, the modes located on the left side of the SPR mode are also highly sensitive [14,25,29] but are narrower, meaning that they can be more easily and accurately monitored through their lower peak [21]. Moreover, the envelope curve can be employed to monitor wavelength shifts of the SPR signature, a method that benefits from the clear attenuation of the cladding modes that couple with the plasmon and is simpler compared to tracking individual resonances [5,30]. A novel approach was recently reported by Lobry et al. [15] based on the intersection point between the lower and upper envelopes. This technique was fully automated, and it considers the whole mode attenuation above and below the SPR attenuation, exhibiting higher sensitivity than the single mode method, though it involves more spectral processing.

In this work, a bare and a plasmonic TFBG were biofunctionalized to perform specific detection of N-terminal B-type natriuretic peptide (NT-proBNP), a biomarker with great relevance for heart failure monitoring. The circulating concentration of this biomarker is increased in heart failure patients, with values in excess of 0.125 ng/mL or 0.300 ng/mL being used, respectively, to rule-in chronic or acute heart failure as a diagnosis [31]. Additionally, NT-proBNP levels also provide prognostic value and allow risk stratification [7]. Furthermore, several spectral analysis techniques were employed and compared to determine the immunosensor performance, namely by tracking the wavelength and/or amplitude shifts of the most sensitive mode and of the spectral envelopes and the envelopes’ crossing point and area.

## 2. Materials and Methods

### 2.1. Reagents

(3-aminopropyl)triethoxysilane (APTES, 98%) and cysteamine hydrochloride (≥98%) were purchased from Sigma-Aldrich, Taufkirchen, Germany. *N*-hydroxysuccinimide (NHS), *N*-(3-dimethylaminopropyl)-*N*′-ethylcarbodiimide hydrochloride (EDC) and hydrogen peroxide (H_2_O_2_, 30% *v*/*v*) were obtained from Merck, Darmstadt, Germany. Sulfuric acid (H_2_SO_4_, 95–97%) was acquired from Fluka, Seelze, Germany. Phosphate buffer saline (PBS, pH = 7.4, 10 mM) tablets were obtained from Fisher Bioreagents, Massachusetts, USA. Bovine serum albumin (BSA) was obtained from Alfa Aesar, Kandel, Germany. Anti-NT-proBNP antibodies (4.72 mg/mL) and NT-proBNP (0.8 mg/mL) were acquired from antibodies-online GmbH, Aachen, Germany. Deionized (DI) water was obtained from a Milli-Q water purification system.

### 2.2. TFBGs’ Fabrication

The TFBGs, 1 cm long, were photo-inscribed in the core of a SMF-28 (Corning Inc., Corning, NY, USA) in order to fabricate the biosensors. This is a single-mode optical fiber with a diameter of 125 μm and a core of 8 μm that was hydrogenated prior to the inscription to enhance photosensitivity. The inscription procedure was based on the phase-mask technique involving the NORIA manufacturing system (Northlab Photonics, Nacka, Sweden), embedding an Argon Fluor deep UV-pulsed excimer laser (Excistar XS, Coherent Inc., Santa Clara, CA, USA), with a 500 Hz repetition rate, emitting at 193 nm, a phase-mask of 1100 nm period and the fibers with an 8° tilt angle with respect to the UV beam axis.

### 2.3. Bare TFBG Immunosensor’s Production

The surface of the bare TFBG was functionalized by firstly yielding free amine groups in order to covalently immobilize, in a second step, the biorecognition molecules, i.e., anti-NT-proBNP antibodies. The protocol applied here was adapted from [5,32]. Initially, the silica fiber, placed inside a polytetrafluoroethylene (PTFE) container (200 μL volume capacity), was treated using piranha solution (3:1 volume ratio H_2_SO_4_:H_2_O_2_) during 10 min for surface hydroxylation, and subsequently, it was rinsed thoroughly with DI water to eliminate remaining residues. Then, the hydroxylated TFBG was silanized by incubation in a mixture of ethanol and DI water (70:30% *v*/*v*) containing 5% *v*/*v* APTES. After 1 h, the fiber surface was washed three times with DI water in order to remove unbounded APTES. Lastly, a cure treatment was performed by placing the fiber in a climatic chamber (LabEvent L C/64/70/3, Weisstechnik, Germany) at 120 °C for 20 min.

**Figure 1:**
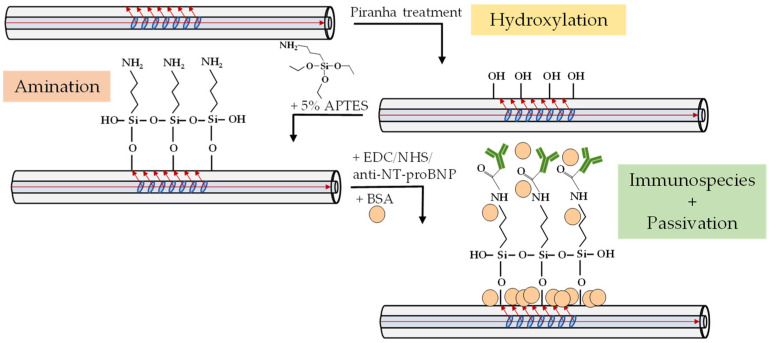
Sketch of the biofunctionalization steps for the bare TFBG.

The amine-terminated fiber was functionalized with anti-NT-proBNP antibodies by being brought in contact for 2 h with a fresh mixture (pH = 7.4) of 100 μL of antibody solution (500 μg/mL), 50 μL of EDC (0.2 M) and 50 μL of NHS (0.5 M), all prepared in PBS solution. Then, the TFBG was washed 3 times with PBS and placed in 200 μL of BSA solution for 2 h to passivate the surface. Thereafter, the fiber was washed with PBS three times and left in this solution overnight. The functionalization steps, which are schematically illustrated in Figure 1, were monitored by collecting optical spectra, with the TFBG immersed in PBS solution, as described in the Section 2.6.

### 2.4. Au–TFBG Immunosensor’s Production

For the Au–TFBG-based immunosensor, after the inscription process described in Section 2.2, a thin Au film, roughly 50 nm thick, was deposited on both sides of the optical fiber in the grating region under vacuum and argon by a sputter-coater (Leica EM SCD 500) in a 2-step process, which involved a 180° rotation of the samples for complete coverage. During the deposition, the thickness of the Au layer was monitored through the in-built quartz microbalance to ensure the desired coating thickness [5].

**Figure 2:**
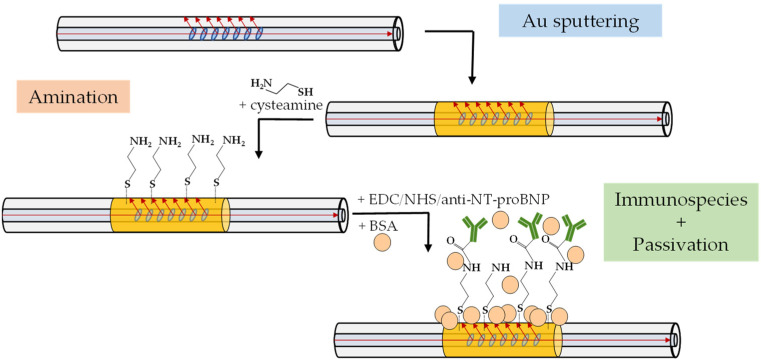
Sketch of the biofunctionalization steps for the Au–TFBG.

The biofunctionalization procedure for the Au–TFBG was performed using cysteamine as the intermediary linker and EDC/NHS chemistry to covalently bind the anti-NT-proBNP antibodies. The protocol followed here was adapted from [5]. Firstly, the fiber was positioned in a PTFE container and cleaned through immersion in an aqueous solution of 10% ethanol for a few minutes. Subsequently, the amine-terminated fiber was obtained by incubating it overnight in an aqueous solution of cysteamine (20 mM, 200 μL). After the incubation time, the Au–TFBG was washed with DI water three times to remove unbounded cysteamine, before being placed in PBS. Similar to the biofunctionalization of the bare TFBG, as described in Section 2.3, the Au–TFBG was immersed for 2 h in a fresh mixture (pH = 7.4) of 100 μL of antibody solution (500 μg/mL), 50 μL of EDC (0.2 M) and 50 μL of NHS (0.5 M), all prepared in PBS. Then, before and after the surface being passivated with BSA solution (10 μg/mL, prepared in PBS) for 2 h, the fiber was washed 3 times in PBS. The functionalization steps (Figure 2) were monitored by recording the optical spectra, with the Au–TFBG immersed in PBS solution, as described in Section 2.6.

### 2.5. NT-proBNP Detection

The functionalized immunosensors were kept inside the PTFE container after the functionalization process and were ready to be tested using different NT-proBNP solutions in the concentration range from 0.01 to 1000 ng/mL. The concentration 1000 ng/mL was prepared from the stock solution, and then successive dilutions of NT-proBNP solution using PBS were made to obtain the concentrations of 0.01, 0.1, 1, 10 and 100 ng/mL. This wide concentration range includes the clinical values of interest for heart failure since values in excess of 0.125 ng/mL or 0.300 ng/mL are used, respectively, to rule-in chronic or acute heart failure as a diagnosis [31].

NT-proBNP detection was performed at room temperature, which was monitored during the course of the experiment, by placing the sensor in analyte solution for 30 min. Thereafter, the TFBG was washed twice in PBS prior to acquisition of the optical transmitted spectrum in PBS solution. This procedure was repeated for every concentration in increasing order to safeguard the availability of binding sites.

### 2.6. TFBGs’ Spectral Acquisition and Data Analysis

**Figure 3:**
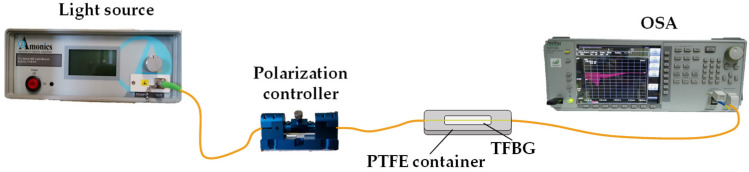
Experimental setup using TFBG sensors (image not to scale).

**Figure 4:**
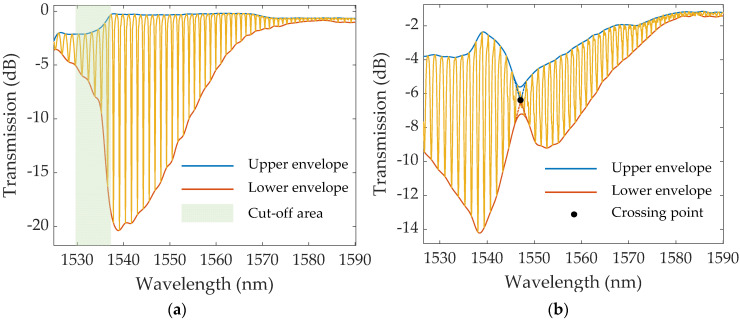
Transmitted spectra denoting the upper and lower envelopes used for the respective methods of analysis for (**a**) the bare TFBG and (**b**) the Au–TFBG.

The TFBGs’ interrogation setup, presented in Figure 3, employed a light source (C + L band ASE light source from Amonics, Kowloon, Hong Kong) that operated in the range of 1528–1608 nm and was connected to a manual polarization controller (FPC-100 from OZ Optics, Ottawa, ON, Canada). The optical spectra of the TFBGs were acquired in the transmission mode, using an optical spectral analyzer (OSA, model MS9740A from Anritsu Corporation, Kanagawa, Japan), with a resolution of 0.05 nm and a wavelength window in the range 1525–1590 nm, connected to the TFBG, which was in turn connected to the polarization controller. In the case of the Au–TFBG, the position of the polarization controller was regulated so that the incident light was P-polarized to enable SPR and further optimized to achieve the highest pinch at the optical spectrum (Figure 4) [5].

The obtained spectra were then studied using MATLAB R2021a, with the methods of spectral analysis used being presented in Figure 4. These methods include (i) tracking the wavelength and amplitude shifts of the most sensitive mode; (ii) monitoring the changes of the lower envelope, as well as (iii) of the upper envelope; (iv) in the case of the Au–TFBG, computing the upper and lower envelopes’ crossing point; and (v) calculating the area between the upper and lower envelopes using the trapz function in MATLAB after normalizing the transmitted spectra to 0–1 dB, in the spectral window of 1530 to 1580 nm. The envelopes were obtained by the envelope function in MATLAB using the “peak” parameter, which applies a spline over local maxima separated by at least *N* points. This value was determined for each TFBG aiming for the most suitable fit to the spectrum (*N* = 900 for bare TFBG and *N* = 1000 for Au–TFBG). The methodology used to calculate the crossing point was based on the one described in [15]. Figure 4a,b exemplify the lower and upper envelopes for a bare TFBG and an Au–TFBG, respectively. Note that in Figure 4a,b, the cut-off region for the bare TFBG and the intersection between the lower and upper envelopes for a given spectrum of the Au–TFBG are highlighted, respectively. 

The performance of the immunosensors and of the demodulation methods were assessed and compared based on the LOD obtained for each case. This figure of merit was determined through the following expression [2]:

(1) $$\mathrm{LOD}=\frac{3\sigma }{S},$$

in which *σ* is the standard deviation of the y-intercept and *S* corresponding to the sensitivity, i.e., the slope of the linear regression obtained for the wavelength or amplitude variations as a function of NT-proBNP concentration, in the linear region of the response.

## 3. Results

The performance of the biosensors for detection of NT-proBNP was evaluated by monitoring the spectral changes in response to immunorecognition events between the biofunctionalized TFBG surface and the peptide. The results obtained with the bare TFBG are presented in Section 3.1, followed by the results attained with the Au–TFBG, which are shown in Section 3.2. 

### 3.1. Bare TFBG

**Figure 5:**
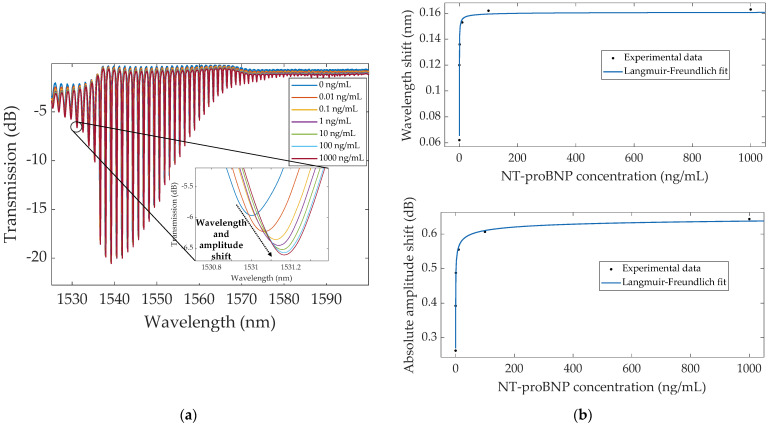
(**a**) Spectral evolution of the biofunctionalized bare TFBG, in PBS, before (0 ng/mL) and after incubation in each concentration of NT-proBNP (0.01–1000 ng/mL), zooming in on the most sensitive mode around 1531 nm; (**b**) Wavelength (top) and absolute amplitude (bottom) variations with concentration, showing the Langmuir–Freundlich fit to the experimental data.

The overlapped spectra of the bare TFBG, each one acquired with the fiber immersed in PBS upon incubation in each concentration of NT-proBNP, are displayed in Figure 5, with focus on the most sensitive mode, around 1531 nm. The resonance chosen for this analysis belongs to the cut-off region, and it was established by closely examining the total wavelength and amplitude shifts of each mode in this region and selecting the one that showed the greatest variations. Similar wavelength shifts were recorded between resonances within the cut-off region, with the most sensitive mode garnering a total wavelength redshift of ~163 pm and a total amplitude increase of 0.64 dB. The most significant shifts of both wavelength and amplitude occurred after contacting with the lower biomarker concentrations, which represent the first binding reactions to the TFBG surface at a moment when every antibody was free to interact with the analyte. As the NT-proBNP concentration increased, fewer binding sites remained available, and thus shorter spectral variations were verified, approaching saturation beyond 100 ng/mL. As such, the immunosensor response could be modeled by the Langmuir–Freundlich isotherm, a mathematical model that considers the interaction between analyte and ligand binding sites, and hence accounts for the saturation effect [33], which is expressed by the following equation:

(2) $$\Delta R=\Delta R_{max}\frac{{\left(KC\right)}^{n}}{1+{\left(KC\right)}^{n}},$$

where Δ*R* is the sensor response (wavelength or amplitude variation) as a function of concentration, *C*; Δ*R_max_* is the maximum response value at the saturation level; *K* is a constant related to the affinity between anti-NT-proBNP antibodies and NT-proBNP antigens; and *n* refers to the index of heterogeneity [33]. 

The wavelength and amplitude variations determined by the single mode method are represented in Figure 5b, evidencing an adequate fit of the Langmuir–Freundlich model to the experimental data. To be able to more easily compare both variables, the absolute values for amplitude shifts were considered.

**Figure 6:**
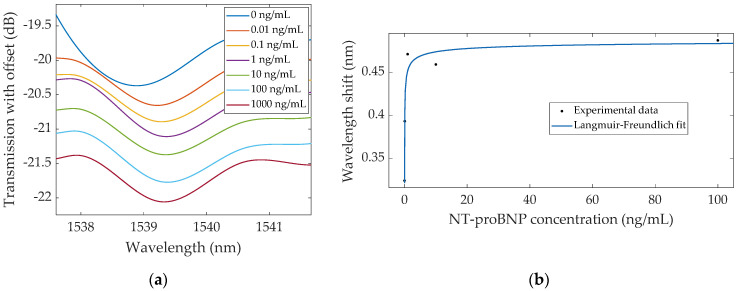
(**a**) Evolution of the lower envelope minimum from the biofunctionalized bare TFBG, recorded after each NT-proBNP concentration; (**b**) Respective wavelength shifts of the lower envelope, evidencing the Langmuir–Freundlich fit.

The other method of spectral analysis consisted of adjusting the lower and upper envelope curves to the spectra and monitoring wavelength shifts. Given the characteristics of the upper envelope, there was not a specific region with clear variations, not exhibiting any tendency with increasing NT-proBNP concentration, and thus the focus was directed at the lower envelopes. The lower envelopes corresponding to each concentration are presented in Figure 6, with an amplitude offset to better visualize the variations. Using this method, spectral changes were decoded through the lower envelope minimum, located near 1539 nm, as it was the easiest point to track and demonstrated clear wavelength shifts, particularly for the lowest NT-proBNP concentrations. A total redshift of ~487 pm was observed after the 100 ng/mL concentration, after which a small decrease was identified following the 1000 ng/mL concentration (~433 pm total increase). The sensor response using this method was properly described by the Langmuir–Freundlich model (Equation (2)), only in the 0.01–100 range given the slight decrease after 100 ng/mL.

The area between the envelopes of the normalized spectra did not evidence a significant variation through increasing concentration, remaining nearly constant.

Finally, two LOD values were calculated using Equation (1) from the linear regions of the wavelength shift as a function of analyte concentration, which was verified for the smaller concentrations, based on the most sensitive mode and the lower envelope. The amplitude shift was not considered to determine the LOD since there was no linear relationship in the experimental data. For the single mode method, the linear region occurred in the 0.1–10 ng/mL range, resulting in an LOD of 7.85 ng/mL, whereas, for the envelope method, a significantly smaller LOD of 0.75 ng/mL was obtained in the 0.01–1 ng/mL range.

### 3.2. Au–TFBG

**Figure 7:**
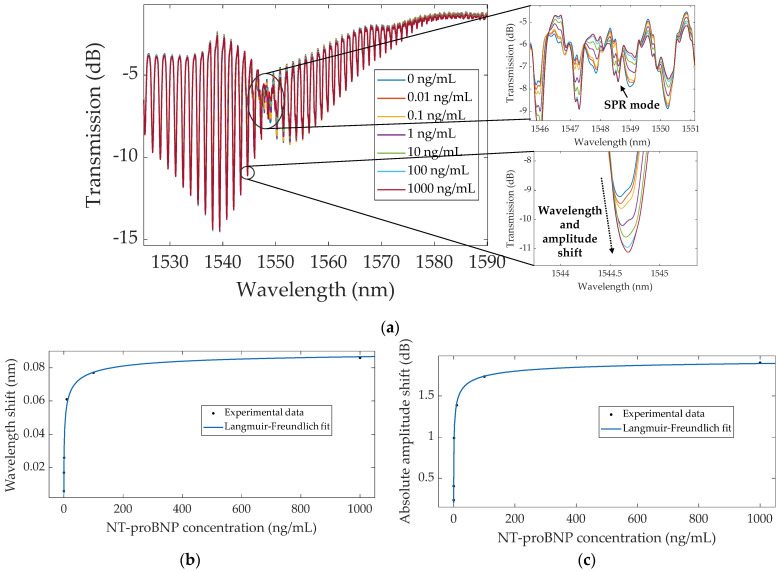
(**a**) Spectral evolution of the biofunctionalized Au–TFBG, recorded in PBS, before (0 ng/mL) and after incubation in each concentration of NT-proBNP (0.01–1000 ng/mL), zooming in on the SPR mode (top inset) and the most sensitive mode around 1544 nm (bottom inset); (**b**) wavelength and (**c**) absolute amplitude shifts as a function of concentration, with the Langmuir–Freundlich fitting to the data.

The same methods of analysis were employed for the Au–TFBG, whose spectral response to different NT-proBNP concentrations are represented in Figure 7. The first method was based on tracking the most sensitive mode, as it was performed with the bare TFBG. As stated in Section 1, despite the SPR mode being highly sensitive, it is too attenuated and broadened to be monitored, as can be seen in the top inset in Figure 7a. Therefore, the modes on the left side of the SPR, bottom inset in Figure 7a, were investigated. It was verified that the total wavelength shifts increased with decreasing distance from the SPR (around 1549), as verified in [28], being of ~43 pm, ~60 pm and ~86 pm for modes around 1542 nm, 1543 nm and 1544 nm, respectively. A similar trend was observed for the amplitude variation: ~1.16 dB, ~1.68 dB and ~1.91 dB for the same resonances. As such, detection was achieved by following the mode at around 1544 nm, which exhibited a 1.91 dB total amplitude increase, which is visually more prominent than the wavelength shift, as verified in [24,29], where biodetection was based solely on amplitude shifts using the most sensitive mode. In this case, the wavelength and amplitude variations of the most sensitive mode were also properly modeled by the Langmuir–Freundlich isotherm, as presented in Figure 7b,c, respectively.

**Figure 8:**
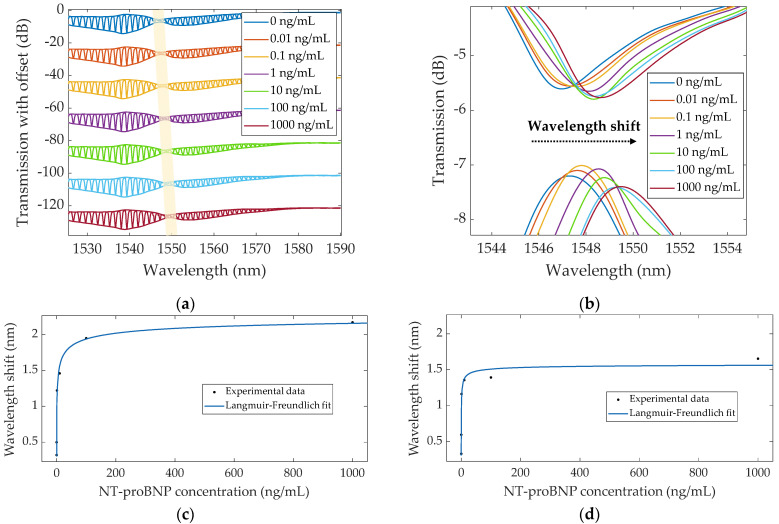
(**a**) Evolution of the spectra and respective envelopes from the biofunctionalized Au–TFBG in response to increasing NT-proBNP concentration, where the shaded area indicates the SPR signature; (**b**) Progression of the lower envelope maximum and upper envelope minimum; Plot of the wavelength shift of (**c**) lower and (**d**) upper envelopes, showing the Langmuir–Freundlich fitting.

The Au–TFBG immunosensor response was then evaluated through envelope analysis. Figure 8a displays the evolution of the envelope curves through increasing NT-proBNP concentration, with the SPR signature highlighted. Following the local maximum of the lower envelope (Figure 8b), there was a total wavelength redshift of ~2.17 nm, a value considerably superior to the redshift determined using the single mode (~86 pm). As for the upper envelope minimum (Figure 8b), a ~1.65 nm total redshift was verified. As observed for the single mode method, the wavelength increase was largest for the lowest concentrations and reduced for the highest, leaning to saturation after 10 and 100 ng/mL for the upper and lower envelopes, respectively, as observed with the Langmuir–Freundlich fitting in Figure 8c,d.

Furthermore, the upper and lower envelopes’ crossing points were computed for each acquired spectrum. With increasing concentration, this point presented a ~1.96 nm total redshift, evidencing saturation beyond 10 ng/mL. 

The area between envelopes revealed an increasing tendency through each NT-proBNP concentration, unlike the bare TFBG, garnering a total increase of ~0.25 arbitrary unit (arb.unit.). In this case, the response did not reach saturation in the range that was tested, leaning towards 0.33 arb.unit.

**Figure 9:**
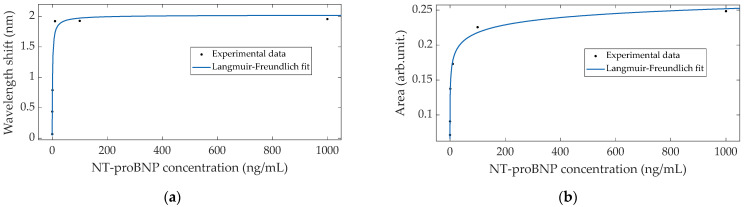
(**a**) Crossing point and (**b**) area variations as a function of NT-proBNP concentration, with the respective Langmuir–Freundlich fittings.

The Langmuir–Freundlich model, expressed by Equation (2), showed an adequate fit to the response observed for each of the demodulation methods, as depicted in Figure 7, Figure 8 and Figure 9, since, in every case, the spectral variations were more pronounced after the first concentrations and eventually reached saturation after the highest ones.

Finally, the LODs were determined for each method using Equation (1) based on the linear region of the spectral response, as explained in Section 2.6. 

## 4. Discussion

The two immunosensors prepared in this work were appropriately functionalized to carry out NT-proBNP detection, with the method chosen for spectral analysis demonstrating a clear impact on their performance. A summary of the results obtained with bare and plasmonic TFBGs is displayed in Table 1, distinguishing the methods of analysis to ease their comparison.

Every demodulation strategy applied in this study was capable of decoding spectral changes for the TFBGs-based immunosensors and were adequately fitted by the Langmuir–Freundlich adsorption model. This model shows that the immunosensor response was largest for the first concentrations tested and diminished with increasing concentration due to saturation, as expected. Nonetheless, the lower envelope method detected a larger response in wavelength for both TFBGs, as is apparent considering the shift at saturation level (Δ*R_max_*), and also reached the lowest LOD values. It is worth noting that, for both TFBGs, the LOD obtained using the lower envelope method is an order of magnitude lower than the one determined with the most sensitive mode method (7.85 vs. 0.75 ng/mL and 1.97 vs. 0.19 ng/mL, respectively). The fact that the wavelength shifts of the envelope curves are superior compared to those of the most sensitive modes might be justified because they take into account the variation of a certain region of the spectrum composed of several resonances that each shift differently, whereas the mode method only tracks the progression of a single resonance and hence is not influenced by its spectral vicinity. Still, this approach provided steady amplitude changes that could corroborate the wavelength shifts, reinforcing the detection results. In fact, for the Au–TFBG, the LOD calculated based on the amplitude shifts was very close to the one obtained with the lower envelope method, supporting this result. However, the amplitude-based methods are more susceptible to external disturbances [23], such as power-level fluctuations.

For the Au–TFBG, the total response of the crossing point was nearly the average of the lower and upper envelope responses since both were considered using this method. Still, for the lowest concentrations, smaller variations were recorded using the crossing point, leading to a higher LOD than the other two methods. 

Looking at the areas between envelopes, there was a successive increase for the Au–TFBG, while there was a negligible variation for the bare TFBG. This result may stem from the greater response in the amplitude of the plasmonic sensor (1.99 vs. 0.67 dB). Furthermore, this method demonstrates better results upon large increases in the surrounding RI since the induced smoothing of the spectrum significantly alters its shape and consequently the area between envelopes. 

It is important to highlight that, due to the large number of cladding mode resonances within the TFBG spectrum, performing spectral analysis is challenging for any method. This is because, firstly, different modes behave distinctly in terms of wavelength and amplitude, and second, because it is almost impossible for the envelope curve to accurately represent the changes manifested by every resonance. Additionally, it makes fitting the envelope to the spectrum more challenging as it must include the characteristics of each resonance. The susceptibility of the envelope fit to each spectrum may be partially responsible for the oscillations in the wavelength shifts verified using the bare TFBG for the higher concentrations. 

In brief, it can be concluded that the method chosen for spectral analysis plays a key aspect for the determination of the biosensor performance and could lead to significantly different results. Despite all strategies presenting benefits and drawbacks, as summarized in Table 2, the lower envelope method proved to be the most appropriate for the intended application of this case-study, as it enabled monitoring spectral changes for smaller concentrations, which are clinically more relevant. Moreover, larger detectable changes before saturation are pivotal for biosensing in order to reliably ascertain the presence or absence of analyte. 

On the other hand, evaluating the two types of TFBG immunosensors, it was verified that there was a large disparity in performance for NT-proBNP detection. Examining the LOD values, the plasmonic sensor achieved superior results regardless of the demodulation method, meaning that it can detect this biomarker at lower concentrations than the bare TFBG. Furthermore, comparing the total envelope shifts, expressed by Δ*R_max_*, the Au–TFBG provided larger spectral changes, making it easier to quantify and thus simplify the data processing necessary to determine biomarker concentration. Therefore, it can be concluded that the SPR effect endows the sensing region greater sensitivity to the binding reactions occurring at the surface of the immunosensor. Nevertheless, the Au–TFBG is much more sensitive to the polarization compared to the bare TFBG, with the SPR signature having to be optimized. Upon these results, the Au–TFBG immunosensor is the most favorable for NT-proBNP detection, exhibiting an LOD with clinical relevance for acute heart failure, though it should be improved for the chronic case.

## 5. Conclusions

The research performed in this work intended to study bare and Au-coated TFBGs in addition to demodulation techniques of their spectral response through a case-study for the detection of NT-proBNP, which is important for monitoring and managing heart failure. With every method employed, the spectral shifts were adequately modeled by the Langmuir–Freundlich adsorption model, presenting the largest changes upon the first contacts with the analyte solution, followed by saturation, as available binding sites became scarcer. Tracking the lower envelope provided the best results, attaining an LOD of 0.75 ng/mL for the bare TFBG and of 0.19 ng/mL for the Au–TFBG. Additionally, following amplitude variations in the most sensitive mode of the Au–TFBG spectrum, an LOD of 0.23 ng/mL was achieved, which was comparable with the LOD value obtained with the lower envelope method for this sensor. Nevertheless, the amplitude response is more vulnerable to external variations, being considered less reliable. Based on these results, it was concluded that the Au–TFBG provided a better performance as it was capable of detecting lower NT-proBNP concentrations that are clinically more significant for heart failure.

While TFBG immunosensors deliver satisfactory performance results, spectral changes are challenging to monitor given the large number of resonances in the spectra. Moreover, each cladding mode possesses distinct sensitivities and hence responds differently both in wavelength and amplitude to external RI variations.

The findings achieved during this work demonstrate the great variety of parameters that can be used to decode spectral variations of TFBGs. The methods used in this work for spectral analysis are universal for TFBGs, meaning that they can be used for the detection of different analytes and biomarkers. The parameters can lead to distinct performance outcomes and should be chosen considering the characteristics of the spectrum: for instance, the presence or absence of the SPR signature and the range of variations in the surrounding RI. For this reason, it is imperative to take into consideration the demodulation techniques applied when comparing the performance between distinct TFBG sensors. 

## Figures and Tables

**Table 1:** Summary of the NT-proBNP detection results for bare and Au-coated TFBGs using the different demodulation methods. Results in bold represent the best achieved LODs for each TFBG.

	Demodulation Method	Parameter	Langmuir–Freundlich		Linear Range (ng/mL)	Sensitivity *	LOD (ng/mL)
			ΔR_max_	R^2^			
Bare TFBG	Sensitive mode	Wavelength (nm)	0.16	0.982	0.1–10	2.7 × 10^−3^	7.85
		Amplitude (dB)	0.67	0.998	-	-	-
	**Lower envelope**	**Wavelength (nm)**	**0.49**	**0.959**	**0.01–1**	**1.2 × 10^−1^**	**0.75**
Au–TFBG	Sensitive mode	Wavelength (nm)	0.09	0.989	0.1–10	4.2 × 10^−3^	1.97
		Amplitude (dB)	1.99	0.996	0.01–1	7.2 × 10^−1^	0.23
	**Lower envelope**	**Wavelength (nm)**	**2.35**	**0.983**	**0.01–1**	**8.7 × 10^−1^**	**0.19**
	Upper envelope	Wavelength (nm)	1.59	0.974	0.01–1	7.5 × 10^−1^	0.42
	Upper and lower envelopes	Crossing point (nm)	2.03	0.970	0.1–10	1.4 × 10^−1^	2.57
		Area (arb.unit.)	0.33	0.993	0.01–1	6.1 × 10^−2^	0.37

* Units for sensitivity depend on the parameter evaluated: nm/(ng/mL) for wavelength and crossing point, dB/(ng/mL) for amplitude and arb.unit./(ng/mL) for area.

**Table 2:** Benefits and drawbacks of different methods of analysis [5,15,23,26,29].

Method of Analysis	Parameter of Analysis	Benefits	Drawbacks
Single mode	Wavelength	Less spectral processing	Small sensitivity for low RI range; Many modes to analyze
	Amplitude	Visually more perceptive; High sensitivity	More susceptible to external influence and power fluctuation; Many modes to analyze
Envelope	Wavelength	High sensitivity; Simple monitoring	Adjustment to the spectra
	Area	High sensitivity	More spectral processing; Small sensitivity for low RI range; Adjustment to the spectra
	Crossing point	Fully automated; Considers the variations of both envelopes	More spectral processing; Adjustment to the spectra

## Data Availability

The data presented in this study are available on request from the corresponding author.
